# Neonatal jaundice and its management: knowledge, attitude and practice of community health workers in Nigeria

**DOI:** 10.1186/1471-2458-6-19

**Published:** 2006-01-27

**Authors:** Olusoga B Ogunfowora, Olusoji J Daniel

**Affiliations:** 1 Department of Paediatrics, College of Health Sciences, Olabisi Onabanjo University, P.M.B. 2022, Sagamu, Nigeria; 2 Department of Community Medicine and Primary Care, College of Health Sciences, Olabisi Onabanjo University, P.M.B. 2022, Sagamu, Nigeria

## Abstract

**Background:**

Neonatal jaundice (NNJ) is still a leading cause of preventable brain damage, physical and mental handicap, and early death among infants in many communities. Greater awareness is needed among all health workers. The objective of the study was to assess the knowledge of primary health care workers about the description, causes, effective treatment, and sequelae of NNJ.

**Methods:**

The setting was a local government area i.e. an administrative district within the south-western part of Nigeria. Community health workers in this area were interviewed by means of a self-administered questionnaire which focused on awareness and knowledge of neonatal jaundice and its causes, treatment and complications.

**Results:**

Sixty-six community health workers participated in the survey and male-to-female ratio was 1:5. Their work experience averaged 13.5 (SD 12.7) years. Only 51.5% of the respondents gave a correct definition of NNJ. 75.8 % knew how to examine for this condition while 84.9 % knew at least two of its major causes in our environment. Also, only 54.5 % had adequate knowledge of effective treatment namely, phototherapy and exchange blood transfusion. Rather than referring affected babies to hospitals for proper management, 13.4 %, 10.4 % and 3 % of the participants would treat with ineffective drugs, natural phototherapy and herbal remedies respectively. None of the participants knew any effective means of prevention.

**Conclusion:**

Primary health care workers may have inadequate knowledge and misconceptions on NNJ which must be addressed concertedly before the impact of the condition on child health and well-being can be significantly reduced. We recommend regular training workshops and seminars for this purpose.

## Background

Primary health care workers are the closest health care providers to the community. By virtue of the location of their duty-post at the periphery of health care delivery network, they serve the important function of 'triaging' patients at the first point of call. With regard to pediatric patients, they are trained to treat minor ailments, administer vaccinations, refer very ill patients to bigger hospitals (2° and 3° care levels) and provide health education to carers of the children among other things. Furthermore, due to the shortage of medically qualified personnel in developing countries, they are given the added responsibility of heading primary health centers.

Neonatal morbidity and mortality remain very high in the developing countries of sub-Saharan Africa, Asia and Latin America [1], and one of the important contributors to this is neonatal jaundice (NNJ) [2-4]. Jaundice due to unconjugated hyperbilirubinemia is also the most common clinical problem in the neonatal period in many parts of the world [2,5-8]. NNJ causes brain damage (kernicterus) when severe [9], leading to neurological handicap and early death of affected infants. Fortunately however, these complications can be avoided by the appropriate use of phototherapy and exchange blood transfusion to control serum bilirubin levels. In order to achieve this, primary health care workers must be able to recognize the condition and refer affected babies to the right places for prompt care and management. More often than not, action taken by these care providers is influenced not only by their knowledge, but also by their perception of, and their attitude to the condition. We are not aware of any previous study that appraised health workers' knowledge on the subject of NNJ.

The present study was therefore designed to assess the knowledge, perception, attitude and practice of primary health care workers in regard to NNJ with a view to identifying any misconceptions and wrong attitude.

## Methods

The study was carried out at the primary health facilities located within Sagamu Local Government Area (LGA) of Ogun State of Nigeria. Out of a total number of twelve, six health centres were randomly selected for the survey by drawing lots. Community health workers at these centers were given self-administered questionnaire to respond to, after obtaining their consent. They were not required to disclose their identity. Medical doctors were excluded from the study.

The survey instrument, designed by the authors, consisted of a 17-item questionnaire [see Additional file 1]. The first part dealt with job designation and experience, and certain demographic data of respondents while the remaining part featured questions on the topic of NNJ. Five questions were open-ended while the remaining ones were of the multiple-choice type. The questions aimed to assess the knowledge of the health workers with regard to definition or description of neonatal jaundice, its causes, treatment and complications and also how to examine the newborn for the presence of NNJ. Question 12 specifically aimed to determine the attitude and practice of the health workers in respect of NNJ.

The primary health care workers were approached individually and informed of the purpose of the survey. Those that gave consent to participate in the study were requested to fill the questionnaire on their own. They were encouraged to seek clarification on any part of the questionnaire from the supervisors but inter-respondent communication was prohibited during the exercise. Four medical students in their penultimate year were given the task of overseeing the exercise.

Data processing and analysis was conducted using Epi-Info version 6.04

## Results

Sixty-six (93%) of 71 health workers in the six randomly-selected community health facilities agreed to participate in the survey. The 5 others withheld their consent for undisclosed reasons. The age of the participants ranged from 25 to 53 yrs with a mean of 36.3 (SD 7.1) yrs and a male-to-female ratio of 1:5. Twenty -four (36.4%) of them were Community Health Officers (CHO) while 29 (43.9%) were Senior Community Health Extension Workers (SCHEW). Other staff cadres represented include Nursing Officers – 4 (6.1%) and Community Health Extension workers (CHEW) – 7 (10.6%). Two respondents did not indicate their designation. Table 1 shows further that the mean number of years of experience of the health workers was 13.5 (SD 12.7).

Thirty-four (51.5%) respondents defined NNJ correctly as yellowish discolouration of the eyes and skin of a baby in the first month (or twenty-eight days) of life due to bilirubin accumulation. 26 (39.4%) responses were either partially correct or incorrect while there was no response from six people. On the question of examining a baby for NNJ, 50 (75.8%) of the respondents chose the three correct options namely;

1-by examining the eyes

2-by looking at the skin and

3-by looking at the palm or sole of the foot.

The remaining 16 (24.2 %) respondents chose 2 of the 3 correct answers.

Question 6 tested the ability of the health workers to recognize signs of danger or complications in NNJ and only 30 (45.5%) were able to choose the six correct answers which were: refusal of feed, high-pitched cry, arching of the back, convulsions, down-turning of the eyes and fever. 19 (28.8%) of them got 5 correct answers right while 6 (9.1%) chose 4 correct answers. Ten (13.1%) respondents however did not know more than one or two of the danger signs.

With regard to common causes of NNJ in our environment, only 25 (37.9%) chose all the three correct answers namely: blood group disparity between mother and child, infection in the baby (sepsis), and prematurity. Thirty-one (47%) respondents and 10 (15.2%) respondents picked two and one correct answer respectively. On the other hand, 23 (34.8%) erroneously chose malaria attack as a cause of NNJ while 6 (9.1%) and 5 (7.6%) chose mosquito bite and germs in the breast milk respectively. On the question of effective treatment for NNJ, 36 (54.5%) of the respondents mentioned both photo therapy and exchange blood transfusion correctly while an additional 21 (31.8%) picked either of the two. Nine health workers (13.6%) did not respond to this question. Conversely, 14 (23.3%) respondents indicated their belief in the efficacy of herbal treatment. The most frequently mentioned herbal remedy was the water extract of unripe paw-paw fruit [9 (64.3%) of 14 respondents]. A large number of respondents, 41 (62.1%), wrongly believed in the effectiveness of certain drugs in the treatment of NNJ, with a majority of them 26 (63.4%), mentioning Ampiclox^® ^syrup, which is a co-formulation of ampicillin and cloxacillin. Only 4 (9.8%) mentioned phenobarbitone. The remaining eleven respondents mentioned a variety of other drugs including chloramphenicol, gentamicin eye drops and vitamins (table 2).

With regard to the management of NNJ, 49 (74.2%) respondents would take the right step by referring affected babies to the hospital immediately, while 9 (13.4%), 7 (10.4%) and 2 (3%) of the respondents would treat such babies with drugs, natural photo therapy and herbal remedies respectively (table 3).

Question 13 tested the awareness of the health workers on the possible complications of NNJ. 66 (100%), 46 (69.7%), 26 (39.4%), 24 (36.4%) and 19 (28.8%) knew that neonatal death, brain damage, cerebral palsy, epilepsy and mental retardation respectively could result from NNJ.

Forty-one (62.1%) respondents have erroneous belief pertaining to prevention of NNJ. Methods of prevention mentioned by them include proper antenatal care – 18 (43.9%) respondents, blood group screening – 12 (29.3%), treatment of malaria in pregnancy – 4 (9.8%), and adequate maternal nutrition in pregnancy – 3 (7.3%). Two respondents (4.9%) thought that yellow fever vaccine could prevent NNJ (table 4).

Finally, 64 (97%) respondents affirmed that they routinely gave health talks on NNJ to pregnant women during antenatal visits, while all 66 thought that training in the management of NNJ would be beneficial to them.

## Discussion

The present study observed a fairly adequate knowledge of the participants in many aspects of NNJ but also revealed some misconceptions and partial knowledge pertaining to certain other aspects which are worthy of note. In the first instance, even though almost all the respondents were aware of the condition and indeed have neonates as clients, but only one half could give a correct definition. Secondly, the participants did not show adequate knowledge of important causes of NNJ in the community where they are serving as health workers. In Nigeria, the leading causes of significant jaundice among neonates include ABO blood group incompatibility, prematurity, sepsis and glucose-6-phosphate dehydrogenase deficiency [10,11]. It was rather surprising that less than half of the participants identified all three of these conditions in the questionnaire. Glucose-6-phosphate dehydrogenase was deliberately left out of the questionnaire on the grounds of being too technical for community health workers to understand.

Another area of inadequate knowledge and misconception observed among some of the respondents in the present study concerns the use of medications in the treatment of NNJ. Whereas more than half of them believed in the effectiveness of certain drugs, only a few mentioned Phenobarbitone, a drug that is known to enhance the conjugation and excretion of bilirubin but which late onset of action and unwanted sedative effect preclude its usefulness in the management of neonatal hyperbilirubinemia [12,13].

Amazingly however and for reasons that are yet obscure, quite a good number of our respondents mentioned Ampiclox^®^, an antibiotic and a co-formulation of ampicillin and cloxacillin, as being effective. We consider this to be a dangerous trend as reliance on unproven medications in the management of NNJ is very hazardous. From our experience, the false sense of security that this practice creates in mothers of affected infants often leads to late presentation of such babies in hospital after kernicterus has already occurred.

We further observed that many respondents in the present study believed in the efficacy of certain herbal remedies for the treatment of NNJ. There is no empirical data to justify this and health care workers need to be persuaded to do away with all wrongly-held traditional beliefs that may affect sound judgment and appropriate discharge of their duties. Phototherapy has remained the standard treatment in neonatal hyperbilirubinemia and exchange transfusion is indicated for severe jaundice [14,15]. These two procedures are widely practiced in our environment and the observation in the present study that some health workers did not have this knowledge is a cause for concern. It is imperative to ensure that community health workers and primary health care practitioners are given adequate exposure to clinical paediatrics during their training.

The health importance of NNJ relates to the neurotoxicity of unconjugated bilirubin. Thus, severely affected babies become brain-damaged and either die or live with serious physical and mental handicap. In this regard, our respondents demonstrated a fairly adequate awareness of some complications of NNJ. Nonetheless, there is a need to reinforce the knowledge of community health workers about other serious conditions such as cerebral palsy, mental retardation, sensori-neural deafness and epilepsy that can result from severe NNJ. This will aid their recognition of NNJ as a potentially serious illness and teach them to take appropriate actions to avoid such complications by promptly referring affected babies to hospital for proper management.

Preventive measures play a minor role in the management of NNJ and without doubt, some of the methods of prevention mentioned by our respondents are definitely not useful for the purpose. These include treatment of malaria in pregnancy, adequate maternal nutrition and yellow fever vaccination. Clinicians therefore need to create a better awareness among primary health care workers of useful preventive measures such as administration of anti- D immune globulin (Rhogam ^®^) to rhesus-negative pregnant women at 28 weeks' gestation or during the immediate post-partum period [16,17]. Health education is an integral part of community health care services and it is heartwarming to observe in our study that all the participants include NNJ in the health talk given to their antenatal clients. It becomes very important that their own knowledge on the subject is regularly updated to ensure that accurate information is passed on to the populace.

## Conclusion

We conclude therefore that knowledge gaps exist among primary health care workers in our locality concerning neonatal jaundice and its management, and we recommend that regular training workshops or seminars be conducted to bridge these gaps. It is believed that this will help to reduce the impact of NNJ on child health and well-being in developing countries of the world.

## Competing interests

The author(s) declare that they have no competing interests.

## Authors' contributions

OBO conceived of the study, drew up the protocol, participated in data analysis and prepared the manuscript. OJD contributed to the study design, supervised data acquisition and conducted data analysis. Both authors read and approved the final manuscript.

## Pre-publication history

The pre-publication history for this paper can be accessed here:



## Supplementary Material

Additional File 1 Questionnaire on neonatal jaundice for health workers. survey instrument. Click here for file

## Figures and Tables

**Table 1:** Demographic and job characteristics of the respondents

Number of respondents	66
Mean (SD) age in years	36.3 (7.1)
Sex ratio : M/F	11/55
Designation :	
No. (%) of CHO	24 (37.5%)
Nursing officers	4 (6.3%)
SCHEW	29 (45.3%)
CHEW	7 (10.9%)
Mean (SD) years of experience	13.5 (12.7)

CHO = Community Health Officer ; SCHEW = Senior Community Health Extension Worker ; CHEW = Community Health Extension Worker

**Table 2:** Drugs believed to be effective in the treatment of NNJ by 41 study participants

**Name of drug**	**No. (%) of respondents**
Ampiclox^®^	26 (63.4)
Phenobarb	4 (9.8)
Vitamins	3 (7.3)
Choramphenicol eye drops	3 (7.3)
Other antibiotics	3 (7.3)
Glucose	2 (4.9)

**Table 3:** What respondents will do with a case of NNJ

**Action**	**No. (%) of respondents**
Refer to hospital	48 (72.7)
Treat with drugs	9 (13.6)
Treat with natural phototherapy	7 (10.6)
Treat with local remedies	2 (3.0)

**Table 4:** Preventive measures mentioned by 41 respondents

**Preventive measure**	**No. (%) of respondents**
Proper antenatal care	18 (43.9)
Blood group screening	12 (29.3)
Treatment of malaria in pregnancy	4 (9.8)
Adequate nutrition in pregnancy	3 (7.3)
Yellow fever vaccine	2 (4.9)
Use of Ampiclox^®^	2 (4.9)
