# Supplementary material for: Neonatal jaundice and its management: knowledge, attitude and practice of community health workers in Nigeria
Source: BMC Public Health. 2006 Jan 27;6:19. doi: 10.1186/1471-2458-6-19 (PMC1409785; doi:10.1186/1471-2458-6-19)
Supplement: Additional File 1 — Questionnaire on neonatal jaundice for health workers. survey instrument. [file 1471-2458-6-19-S1.doc]

QUESTIONNAIRE ON NEONATAL JAUNDICE

FOR HEALTH WORKERS

***Kindly answer the following questions as best as you can. It is not necessary to write your name.***

**BIOGRAPHIC DATA:**

Age in years________________ Sex: M / F Designation (position / rank) _________________ __ __

**Qualification:** Tick the most appropriate

i. University degree [specify] _________________________________________

ii. Public Health Nursing certificate ____________________________________

iii. Community Health Officer certificate _________________________________

iv Registered Nurse/Midwife certificate _________________________________

v . Community Health Technician certificate _____________________________

vi. Any other [specify] _____________________________________________

Years of post-qualification experience______________________________________________

1. How long have you been working at the primary health care level? __________________
2. (a) Do you have newborn babies as clients? Yes / No ____________________________

(b) Have you ever heard of Neonatal Jaundice? Yes / No _________________________

1. If yes, state in simple terms what you understand by the term neonatal jaundice:

______________________________________________________________________________________________________________________________________________

1. How would you check a baby for the presence of jaundice? You can pick more than one answer:
   - 1. By examining the eyes ___________________________
     2. By looking at the skin _______________________________
     3. By looking at the palm or sole of the foot _______________
     4. By the colour of the urine ___________________________
     5. By the colour of the stool ____________________________
     6. By other means [specify] ____________________________
2. When a baby has jaundice, urine colour may be? You can pick more than one answer
   - 1. White ____________________________________
     2. Yellow ____________________________________
     3. Greenish-yellow ____________________________
     4. Any other colour [specify] ______________ _______
3. What are the danger signs in a baby with neonatal jaundice? You can pick more than one answer.
   - 1. Refusal of feed ____________________________
     2. High-pitched cry _____________________________
     3. Arching of the back _____________________________
     4. Convulsions _____________________________
     5. Down-turning of the eyes __________________________
     6. Fever __________________________________________
     7. Any other [specify] _________________________ __ __ _
4. Neonatal Jaundice can be caused by any of the following: (you can pick more than one answer)
   1. Disparity between the blood group of mother and child. ________________
   2. Bacterial infection in the blood (sepsis) _____________________________
   3. Malaria attack. _________________________________________________
   4. Mosquito bite. _________________________________________________
   5. Germs in the breast or breast milk. ________________________________
   6. Prematurity ___________________________________________________
   7. Any other [specify] _____________________ ______ __ _
5. Do you know if any of the following is effective in the treatment of neonatal jaundice? Answer yes or no: i. photo therapy ______________________________________

ii exchange blood transfusion ___________________________

1. Do you know any drug that is effective in the treatment of neonatal jaundice ? Yes / No _____________
2. If yes, mention it (them) i.________________________ ii._____ _____________ ___ __

1. Is there any local treatment that you know to be effective? Yes/No ________________

If yes mention it (them) ________________________________________________

_______________________________________________

1. If a baby with neonatal jaundice is brought to you for treatment, what will you do? Tick the most appropriate answer:
   1. Treat with some drugs __________________________________________
   2. Recommend local remedies ______________________________________
   3. Refer baby to hospital immediately _________________________________
   4. Advise mother to place the baby under the sun _______________________
   5. Take other action [specify] ____________________________ _ __________
2. Do you know whether severe jaundice can have any of the effects mentioned below? You can pick more than one answer.
   1. Death of a baby _____________________________________
   2. Brain damage in the baby _____________________________
   3. Mental retardation __________________________________
   4. Physical handicap ___________________________________
   5. Attacks of convulsion later in life _________________________
   6. Abnormal behaviour later in life ________________________
   7. Other effect[s] [specify] _______________________________
3. Do you know any means of prevention? Yes / No ____________________

1. If yes, describe _________________________________________________

_______________________________________________

1. Do you include neonatal jaundice in the health talk given to antenatal clinic clients? Answer Yes or No _________________________________________________

1. Do you think you need training in the management of neonatal jaundice? Yes / No ____________.

**Thank you very much for taking part in this exercise.**
